# Supplementary material for: High-order dynamic localization and tunable temporal cloaking in ac-electric-field driven synthetic lattices
Source: Nat Commun. 2022 Dec 10;13:7653. doi: 10.1038/s41467-022-35398-9 (PMC9741653; doi:10.1038/s41467-022-35398-9)
Supplement: Supplementary file 1 — Supplementary Information [file 41467_2022_35398_MOESM1_ESM.pdf]

Supplementary Information for

High-order dynamic localization and tunable temporal cloaking in ac-electric-field driven synthetic lattices

Shulin Wang[1,2,#], Chengzhi Qin[1,2,#], Weiwei Liu[1,2,#], Bing Wang[1,2,*], Feng Zhou[1,2], Han Ye[1,2], Lange Zhao[1,2], Jianji Dong[1,2], Xinliang Zhang[1,2], Stefano Longhi[3,4,*], and Peixiang Lu[1,2,5,*]

[1]Wuhan National Laboratory for Optoelectronics and School of Physics, Huazhong University of Science and Technology, Wuhan 430074, China.

[2]Optics Valley Laboratory, Hubei 430074, China.

[3]Dipartimento di Fisica, Politecnico di Milano, Piazza Leonardo da Vinci 32, I-20133 Milano, Italy.

[4]IFISC (UIB-CSIC), Instituto de Fisica Interdisciplinar y Sistemas Complejos, E-07122 Palma de Mallorca, Spain.

[5]Hubei Key Laboratory of Optical Information and Pattern Recognition, Wuhan Institute of Technology, Wuhan 430205, China.

[#]These authors contributed equally to this work

[*]Corresponding authors:

B. W. (email: wangbing@hust.edu.cn),

S. L. (email: stefano.longhi@polimi.it),

P. L. (email: lupeixiang@hust.edu.cn).

Supplementary Note 1: Derivation of band structure for the temporal lattice

In this section, we provide the detailed derivation of the band structure for the synthetic temporal lattice. Without the additional phase modulation, the pulse dynamics in the double fiber-loops is governed by the following equations

$$\begin{cases} u_n^m = \cos(\beta) u_{n+1}^{m-1} + i\sin(\beta) v_{n+1}^{m-1} \\ v_n^m = i\sin(\beta) u_{n-1}^{m-1} + \cos(\beta) v_{n-1}^{m-1} \end{cases}, \tag{1}$$

in which u_n^m and v_n^m represent the complex amplitudes of the pulses in the short and long loops, respectively. The Bloch mode is assumed as

$$\begin{pmatrix} u_n^m \\ v_n^m \end{pmatrix} = \begin{pmatrix} U \\ V \end{pmatrix} e^{iQn} e^{i\theta m}, \tag{2}$$

where $(U, V)^{\mathrm{T}}$ represents the eigenvector. Substituting the Bloch mode into Eq. (1), we can obtain

$$\begin{pmatrix} U \\ V \end{pmatrix} e^{iQn} e^{i\theta m} = \begin{pmatrix} \cos(\beta) e^{iQ} e^{-i\theta} & i\sin(\beta) e^{iQ} e^{-i\theta} \\ i\sin(\beta) e^{-iQ} e^{-i\theta} & \cos(\beta) e^{-iQ} e^{-i\theta} \end{pmatrix} \begin{pmatrix} U \\ V \end{pmatrix} e^{iQn} e^{i\theta m}. \tag{3}$$

Equation (3) can be simplified to an eigenvalue equation

$$e^{i\theta} \begin{pmatrix} U \\ V \end{pmatrix} = \begin{pmatrix} \cos(\beta) e^{iQ} & i\sin(\beta) e^{iQ} \\ i\sin(\beta) e^{-iQ} & \cos(\beta) e^{-iQ} \end{pmatrix} \begin{pmatrix} U \\ V \end{pmatrix}. \tag{4}$$

We then arrive at

$$\begin{vmatrix} \cos(\beta) e^{iQ} - e^{i\theta} & i\sin(\beta) e^{iQ} \\ i\sin(\beta) e^{-iQ} & \cos(\beta) e^{-iQ} - e^{i\theta} \end{vmatrix} = 0. \tag{5}$$

from which we can obtain the band structure

$$\theta_{\pm} = \pm \arccos\left[\cos(\beta)\cos(Q)\right]. \tag{6}$$

We are mainly interested in a splitting parameter β close to π/2. In this case, we can expand the function arccos(x) to the Taylor series, i.e.,

$$\arccos x = -\arcsin x + \frac{\pi}{2} = -\left(x + \frac{1}{2} \times \frac{x^3}{3} + \frac{1}{2} \times \frac{3}{4} \times \frac{x^5}{5} + \ldots \right) + \frac{\pi}{2}, |x| < 1, \tag{7}$$

where x is the function argument. Hence, the band structure can be expanded to

$$\theta_{\pm} = \mp \left\{ \cos(\beta)\cos(Q) + \frac{1}{2} \times \frac{\left[\cos(\beta)\cos(Q)\right]^3}{3} + \frac{1}{2} \times \frac{3}{4} \times \frac{\left[\cos(\beta)\cos(Q)\right]^5}{5} + \ldots \right\} \pm \frac{\pi}{2}. \tag{8}$$

As β is near π/2, the term cos(β)cos(Q) approaches zero. As a consequence, high order terms of Taylor series can be ignored, suggesting that we can approximate the band structure to

$$\theta_{\pm} = \mp\cos(\beta)\cos(Q) \pm \frac{\pi}{2}. \tag{9}$$

As a comparison, we plot the exact and approximate band structures in Fig. S1. For $\beta = \pi/2$, the exact and the approximate band structures are identical because the term $\cos(\beta)\cos(Q)$ equals zero. As depicted in Fig. S1a, the exact and approximate band structures coincide fully. For $\beta = \pi/3$, the approximate band structure is also very close to the exact one, as displayed in Fig. S1b. As $\beta\to0$, the term $\cos(\beta)\cos(Q)$ approaches ±1 when $Q\to0$ or $\pm\pi$, such that high order terms of Taylor series cannot be ignored. As a result, the approximation of band structure does not work well. As displayed in Figs. S1c and S1d, the approximate band structure deviates apparently from the exact one when $Q\to0$ or $\pm\pi$.

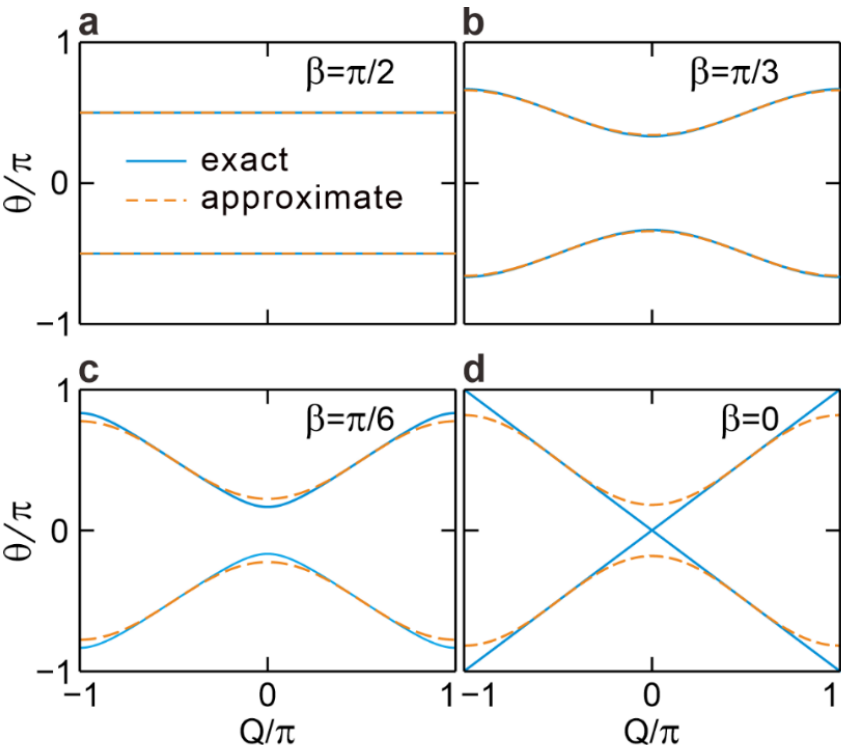

Fig. S1. Band structure of synthetic temporal mesh lattice. a-d Band structures for $\beta = \pi/2$, $\pi/3$, $\pi/6$ and 0, respectively. The solid and dashed curves represent the exact and approximate band structures, respectively.

Supplementary Note 2: Quasi-energy band structure and the Dunlap-Kenkre model

A. Quasi-energy band structure

We consider a sinusoidally varying phase modulation $\phi(m) = \Delta\phi\cos(\omega m+\varphi)$, where $\Delta\phi$, ω and φ denote the amplitude, frequency and initial phase of phase modulation, respectively. The instantaneous band structure is obtained from Eq. (9) after the replacement $Q\to Q-\phi(m)$ and reads

$$\theta_{\pm}(m) = \mp\cos(\beta)\cos\left[Q-\Delta\phi\cos(\omega m+\varphi)\right]\pm\frac{\pi}{2}. \tag{10}$$

In the large M limit, as shown below the quasi-energy bands are simply obtained by averaging the instantaneous band structure within the driving period $M = 2\pi/\omega$, i.e.,

$$\begin{aligned}
\langle\theta_{\pm}\rangle &= \frac{1}{M}\int_0^M \theta_{\pm}(m)dm = \frac{1}{M}\int_0^M\left\{\mp\cos(\beta)\cos\left[Q-\Delta\phi\cos(\omega m+\varphi)\right]\pm\frac{\pi}{2}\right\}dm \\
&= \mp\cos(\beta)\frac{1}{M}\int_0^M\left\{\cos(Q)\cos\left[\Delta\phi\cos(\omega m+\varphi)\right]+\sin(Q)\sin\left[\Delta\phi\cos(\omega m+\varphi)\right]\right\}dm\pm\frac{\pi}{2} \\
&= \mp\cos(\beta)\cos(Q)\frac{1}{\omega M}\int_{\varphi+\frac{\pi}{2}}^{\varphi+\frac{5\pi}{2}}\cos\left[\Delta\phi\sin\left(\omega m+\varphi+\frac{\pi}{2}\right)\right]d\left(\omega m+\varphi+\frac{\pi}{2}\right) \\
&\quad\mp\cos(\beta)\sin(Q)\frac{1}{\omega M}\int_{\varphi+\frac{\pi}{2}}^{\varphi+\frac{5\pi}{2}}\sin\left[\Delta\phi\sin\left(\omega m+\varphi+\frac{\pi}{2}\right)\right]d\left(\omega m+\varphi+\frac{\pi}{2}\right)\pm\frac{\pi}{2}.
\end{aligned} \tag{11}$$

On the other hand, the definition of Bessel function is

$$\begin{cases}
\dfrac{1}{2\pi}\displaystyle\int_{\varphi'}^{\varphi'+2\pi}\sin\left(x\sin\theta'-N\theta'\right)d\theta' = 0, \\
\dfrac{1}{2\pi}\displaystyle\int_{\varphi'}^{\varphi'+2\pi}\cos\left(x\sin\theta'-N\theta'\right)d\theta' = J_N(x),
\end{cases} \tag{12}$$

where N is the order of the Bessel function, φ' and θ' are function arguments. Hence, the quasi-energy band structure can be simplified to

$$\begin{aligned}
\langle\theta_{\pm}\rangle &= \mp\cos(\beta)\cos(Q)J_0(\Delta\phi)\mp\cos(\beta)\sin(Q)\times 0\pm\frac{\pi}{2} \\
&= \mp J_0(\Delta\phi)\cos(\beta)\cos(Q)\pm\frac{\pi}{2}.
\end{aligned} \tag{13}$$

Finally, let us rigorously justify why in the large M limit the quasi-energy bands can be obtained by the averaging method, leading to Eq. (13). We start from the discrete-time coupled equations

$$\begin{cases}
u_n^m = \left[\cos(\beta)u_{n+1}^{m-1}+i\sin(\beta)v_{n+1}^{m-1}\right]e^{-i\phi(m)} \\
v_n^m = \left[i\sin(\beta)u_{n-1}^{m-1}+\cos(\beta)v_{n-1}^{m-1}\right]e^{i\phi(m)}
\end{cases}. \tag{14}$$

Owing to the discrete translational invariance of the lattice, we look for a solution to Eq. (14) in the form of a Bloch wave with transverse wave number Q

$$\begin{cases} u_n^m = U^m e^{iQn} \\ v_n^m = V^m e^{iQn} \end{cases}, \tag{15}$$

where, after setting $x_m=(U^m, V^m)^T$ the evolution of x_m over one discrete time step is governed by $x_m=P_m x_{m-1}$ with the propagator

$$P_m = \begin{pmatrix} \cos(\beta)\exp(iQ_m) & i\sin(\beta)\exp(iQ_m) \\ i\sin(\beta)\exp(-iQ_m) & \cos(\beta)\exp(-iQ_m) \end{pmatrix}, \tag{16}$$

where we have set $Q_m = Q-\phi(m)$. Using the composition law of the SU(2) group, the propagator P_m can be written in terms of the exponential of a Hamiltonian H_m represented by a Pauli vector, i.e. $P_m = \exp(-iH_m)$ with

$$H_m = -\frac{\sin\theta(Q_m)}{\theta(Q_m)} \begin{pmatrix} \cos(\beta)\sin(Q_m) & \sin(\beta)\exp(iQ_m) \\ \sin(\beta)\exp(-iQ_m) & -\cos(\beta)\sin(Q_m) \end{pmatrix}, \tag{17}$$

where we have set

$$\theta(Q_m) \equiv \arccos\left[\cos(\beta)\cos(Q_m)\right]. \tag{18}$$

Note that the eigenvalues of H_m are given by $\pm\theta(Q_m)$, i.e. they are the instantaneous eigen-energies introduced above [Eq. (10)]. Assuming $\phi(m) = \Delta\phi\cos(\omega m+\varphi)$ with a modulation frequency $\omega = 2\pi/M$, in the large M limit $\phi(m)$, and thus Q_m and H_m, vary slowly as m changes by ±1. In this case m can be considered as a continuous variable and the propagator $P = P_M P_{M-1} \ldots P_1$ of the system over one oscillation cycle, i.e. from $m = 0$ to $m = M$, can be written as the ordered exponential of H_m, i.e.

$$P = \mathcal{T}\left\{\exp\left(-i\int_0^M dm H_m\right)\right\}. \tag{19}$$

The quasi-energies (Floquet exponents) $\pm\varepsilon(Q)$ are related to the eigenvalues μ, $1/\mu$ of the matrix P by the relation $\exp(-iM\varepsilon(Q)) = \mu$. In the large M limit, the form of $\varepsilon(Q)$ can be readily obtained by a standard WKB analysis, and reads

$$\varepsilon(Q) = \frac{1}{M}\int_0^M \theta(m)\, dm. \tag{20}$$

This relation provides the justification of the average method used to derive Eq. (11), i.e. $\varepsilon(Q) = \langle\theta(Q_m)\rangle$.

B. Relation to the Dunlap-Kenkre model of dynamic localization

Here we show in details that, for a splitting parameter β close to $\pi/2$ and large M period, the discrete photonic quantum walk setup exactly emulates the original Dunlap-Kenkre continuous model of dynamic localization (DL) for an electron hopping on a tight-binding lattice subjected to an ac sinusoidal electric field. To this aim, let us assume β close to $\pi/2$ and set $\cos\beta = \varepsilon$, $\sin\beta = 1+O(\varepsilon^2)$, where ε is a small parameter. At leading order in ε, the map Eq. (14) takes the form

$$\begin{cases} u_n^m = \left(\varepsilon u_{n+1}^{m-1} + i v_{n+1}^{m-1}\right)\exp\left[-i\phi(m)\right] \\ v_n^m = \left(i u_{n-1}^{m-1} + \varepsilon v_{n-1}^{m-1}\right)\exp\left[i\phi(m)\right] \end{cases}, \tag{21}$$

from which, after elimination of v_n^m, one readily obtains the following discrete equation for u_n^m

$$u_n^{m+1} = -u_n^{m-1}\exp\left[i\phi(m) - i\phi(m+1)\right] + \varepsilon u_{n+1}^m \exp\left[-i\phi(m+1)\right] + \varepsilon u_{n-1}^m \exp\left[2i\phi(m) - i\phi(m+1)\right], \tag{22}$$

where $\phi(m) = \Delta\phi\cos(\omega m + \varphi)$ and $\omega = 2\pi/M$. In the large M limit, $\phi(m)$ varies slowly with m so that we may set $\phi(m+1) - \phi(m) \approx (d\phi/dm) \sim \varepsilon$, etc. After letting $u_n^m = i^m U_n^m$, one then obtains

$$U_n^{m+1} = U_n^{m-1}\exp\left[-i\left(d\phi/dm\right)\right] - i\varepsilon U_{n+1}^m \exp\left[-i\phi(m)\right] - i\varepsilon U_{n-1}^m \exp\left[i\phi(m)\right]. \tag{23}$$

Since ε and $(d\phi/dm) \sim \varepsilon$ are small parameters, this relation indicates that U_n^m varies slowly after two steps, i.e. from $(m-1)$ to $(m+1)$. Hence at leading order one has either $U_n^m = \psi_n(m)$ or $U_n^m = (-1)^m\psi_n(m)$, with $\psi_n(m)$ slowly varying with respect to m. The two cases basically correspond to the two energy bands of the synthetic mesh lattice. Let us focus for the sake of definiteness to the former case, the latter case just corresponding to a sign flip of the energy band. After letting $U_n^m = \psi_n(m)$ with $\psi_n(m)$ slowly-varying function of index m, at leading order in ε from Eq. (23) one obtains

$$i\frac{d\psi_n}{dm} = \frac{1}{2}\left(\frac{d\phi}{dm}\right)\psi_n + \frac{1}{2}\varepsilon\left\{\psi_{n+1}\exp\left[-i\phi(m)\right] + \psi_{n-1}\exp\left[i\phi(m)\right]\right\}, \tag{24}$$

which can be viewed as a continuous photonic quantum walk in the continuous time variable m on a one-dimensional tight-binding lattice with hopping amplitude $(\varepsilon/2)$. Finally, the Dunlap-Kenkre model is readily obtained after the gauge transformation

$$\psi_n(m) = c_n(m)\exp\left[i(n - 1/2)\phi(m)\right], \tag{25}$$

yielding the following coupled equations for the wave amplitudes $c_n(m)$

$$i\frac{dc_n}{dm} = \frac{1}{2}\varepsilon\left\{c_{n+1} + c_{n-1}\right\} + F(m) n c_n, \tag{26}$$

where

$$F(m) = \frac{d\phi}{dm} = -\omega\Delta\phi\sin(\omega m + \varphi) \tag{27}$$

is the ac driving electric force. The condition for quasi-energy band collapse, i.e. DL, as obtained from the Dunlap-Kenkre model with the electric force given by Eq. (27), is clearly in agreement with the condition Eq. (20) based on the average of instantaneous bands of the synthetic mesh lattice in the $\beta \to \pi/2$ limit.

C. Exact quasi-energy spectrum under strong driving

The scenario for high field amplitudes is very different when dealing with a discrete-time photonic

quantum walk system, as compared to the usual scenario found in continuous-time ac-driven systems. The photonic quantum walk on a mesh lattice basically reduces to the continuous-time Dunlap-Kenkre model in the adiabatic limit of slow modulation, where the phase $\phi(m) = \Delta\phi\cos(\omega m+\varphi)$ varies slowly with the discrete time index m. In our experiment we assumed in fact a low oscillation frequency ($\omega = \pi/60$). For increasing values of the field amplitude $\Delta\phi$, one can qualitatively distinguish three different regimes: (i) For low-to-moderate modulation amplitudes, the continuous-time limit of the dynamics is valid and the ac field does not couple the two bands. In this regime we have DL like in the usual single-band Dunlap-Kenkre model, with quasi-energy band collapse observed at specific values of $\Delta\phi$, corresponding to the roots of the J_0 Bessel function. (ii) For moderately high values of modulation amplitude, the coupling of two bands occurs and Zener tunneling is not negligible anymore. The quasi-energy band collapse is imperfect. (iii) At very high modulation amplitudes $\Delta\phi$, such that $\Delta\phi\omega$ ceases to be smaller than 1, even for a slow oscillation frequency the phase $\phi(m)$ does not change slowly with the index m, and thus the discrete nature of temporal evolution cannot be disregarded anymore. A remarkable feature of the discreteness of time evolution in this regime is the absence of quasi-energy band flattening known for continuous-time two-band ac-driven systems. As a matter of fact, in the ultrastrong amplitude regime we need to numerically compute the exact quasi-energy bands of the discrete-time mesh lattice. Figure S2 shows, as an illustrative example, the behavior of quasi-energies of the discrete-time mesh-lattice versus modulation amplitude $\Delta\phi$ for parameter values $\omega = \pi/30$, $\beta = 0.97\times\pi/2$, as obtained from the exact numerical computation of Floquet exponents of the one-period propagator of the system (Fig. S2b), and from the asymptotic relation $\varepsilon(Q) = -J_0(\Delta\phi)\cos(\beta)\cos(Q)+\pi/2$ corresponding to the averaging method discussed in the main manuscript (Fig. S2a). For the sake of clearness, only the upper quasi-energy band is depicted in the figure. Note that, for low to intermediate values of the modulation amplitude $\Delta\phi$, quasi-energy band collapse at the various DL orders (zeros of J_0 function) is observed, in agreement with the Dunlap-Kenkre model. However, at high values of the modulation amplitude $\Delta\phi$, quasi-energy band collapse in not exact (see for example the case $\Delta\phi = 30.6$, corresponding to the 10th root of Bessel function, shown in the right panels of Fig. S2). Remarkably, unlike the Dunlap-Kenkre model the width of the quasi-energy band does not shrink to zero at very high values of the field amplitude $\Delta\phi$, and displays an irregular behavior (Fig. S2b). Remarkably, the width of the quasi-energy band can even increase, on average, as $\Delta\phi$ increases, as clearly shown in Fig. S2b for amplitudes $\Delta\phi$ larger than 60. This means that, unlike continuous-time ac driven systems, in discrete-time ac-driven systems the dynamics is not frozen and delocalization seems to be ubiquitous in the ultrastrong field amplitude regime. This is a strict distinctive feature of the DL in the photonic quantum walk system arising from the discrete nature of time evolution, which does not have any

counterpart in the classic model of DL in continuous-time systems.

For real applications, where noise and/or imperfections in the system are unavoidable, the most stable operational regime is surely the moderate-to-high-order DL regime, i.e., typically from 3rd to 6th orders. Here the localization condition is very tight and more robust against noise than low-order DL regime. However, one should avoid pushing the DL resonance condition to very high orders. In fact, in this case the discrete nature of the dynamics prevents collapse of the quasi-energy band, and thus localization, even in the absence of any external noise.

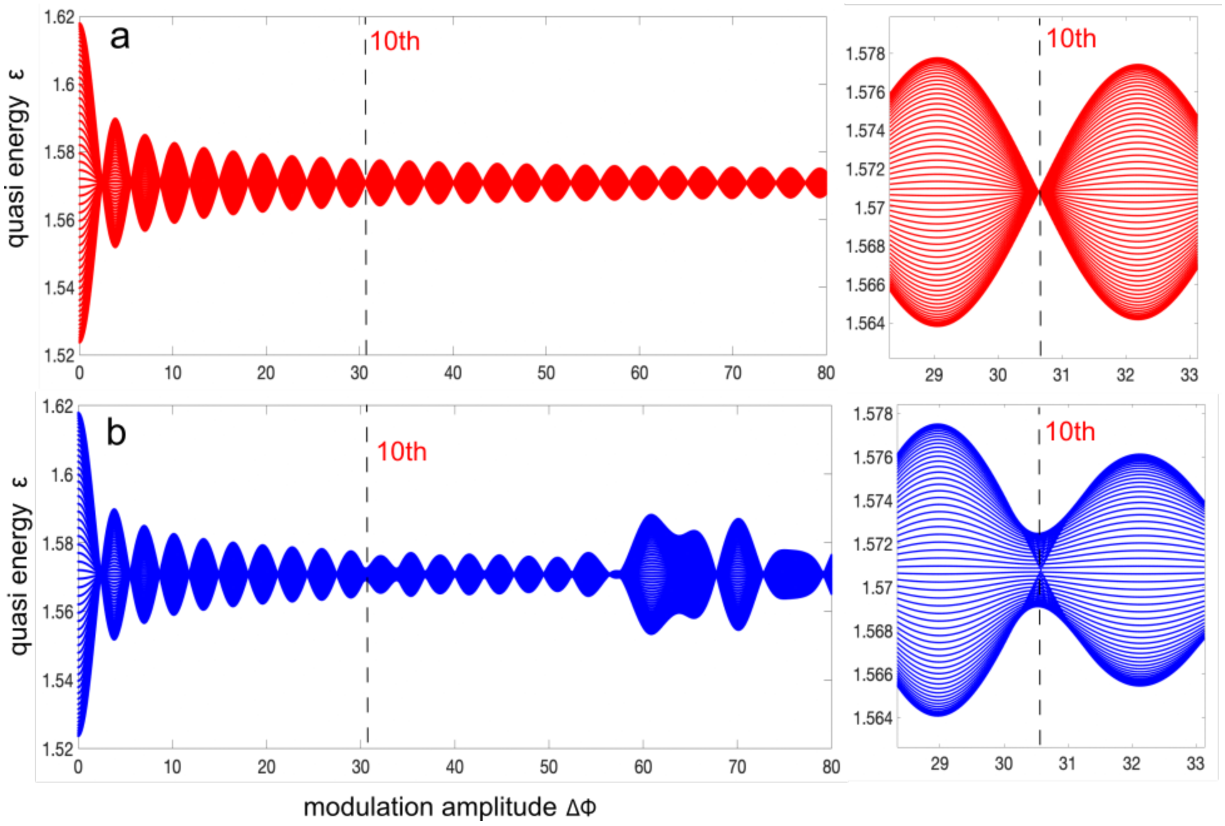

Fig. S2. a Quasi-energy spectrum $\varepsilon(Q)$ versus modulation amplitude $\Delta\phi$ calculated by the average method under the continuous-time limit. **b** Exact quasi-energy spectrum obtained from numerical computation. The parameter values are $\omega = \pi/30$, $\beta = 0.97\times\pi/2$. Only the upper quasi-energy band is plotted. The right panel shows an enlargement of the quasi-energies near the 10th root of J_0 Bessel function (vertical dashed line).

Supplementary Note 3: Mean-square displacements and pulse intensity evolutions of different-order DLs

Figure S3 illustrates the mean-square displacements and pulse intensity evolutions of the 1st- to 5th-order DLs. As displayed in Figs. S3b and S3c, the higher-order DL has smaller oscillation amplitude and thus stronger localization strength over the lower-order one. We then depict the evolution of mean-square displacement $\langle n^2(m)\rangle$ for various-order DLs in Fig. S3a. Considering the periodic oscillation of wave packet, the mean-square displacement $\langle n^2(m)\rangle$ also exhibits periodic oscillations during propagation. From the 1st- to 5th-order DLs, the maximum of mean-square displacement $\langle n^2(m)\rangle_{max}$ decreases from 48.1 to 14.9, further demonstrating the better localization strength of higher-order DL.

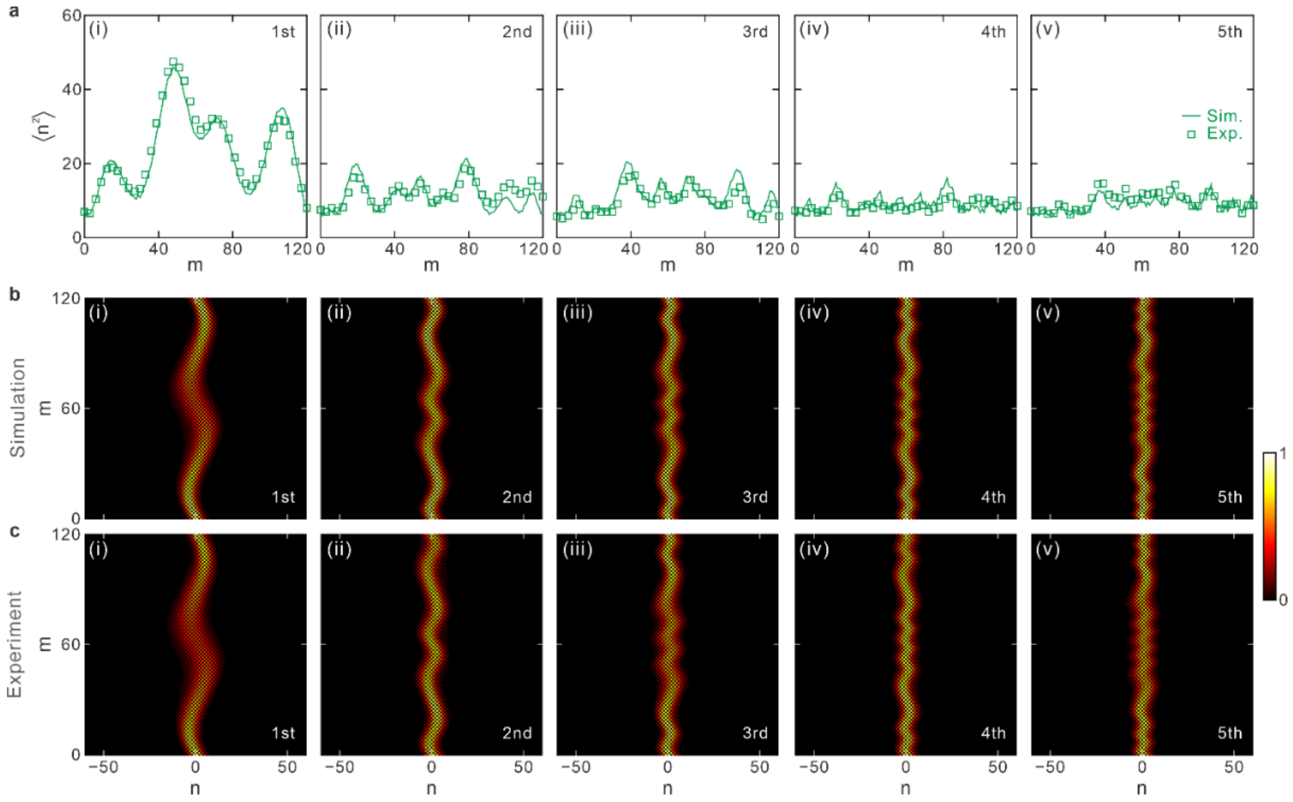

Fig. S3. Mean-square displacements and pulse intensity evolutions of various-order DLs. a Mean-square displacement $\langle n^2(m)\rangle$ varying with step m for different orders of DLs. (i)-(v) correspond to the 1st- to 5th-order DLs. **b** Simulated pulse intensity evolutions. (i)-(v) correspond to the 1st- to 5th-order DLs. **c** Measured pulse intensity evolutions. (i)-(v) correspond to the simulated results in **b**(i)-**b**(v).

Supplementary Note 4: Explanation for enhanced localization strength of high-order DL

A simple physical explanation of the enhanced localization strength of high-order DL can be gained by analyzing the instantaneous wave-packet dynamics in both momentum and real spaces. For a Gaussian wave packet in the real space, the corresponding distribution in the momentum space also has a Gaussian envelope centered at a certain momentum. Approximately, the wave packet can be treated as a quasi-particle with certain position and momentum simultaneously. Under the artificial ac electric field, the mean momentum experiences periodic variation according to $Q(m) = Q - \Delta\phi\cos(\omega m+\varphi)$, as shown in Figs. S4a and S4b. During the variation, as the instantaneous momentum $Q(m)$ reaches the boundary and center of Brillouin zone, the sign of group velocity v_g flips, suggesting the Bragg reflection of wave packet in real space. For the higher-order DL, the larger electric field can drive the Bloch wave packet to oscillate across a larger regime in the extended Brillouin zone, leading to more times of packet Bragg reflections at each Brillouin zone boundary and center. More frequent Bragg reflections will cancel the net accumulated packet motion in one direction within a driving period and hence give rise to the stronger localization strength.

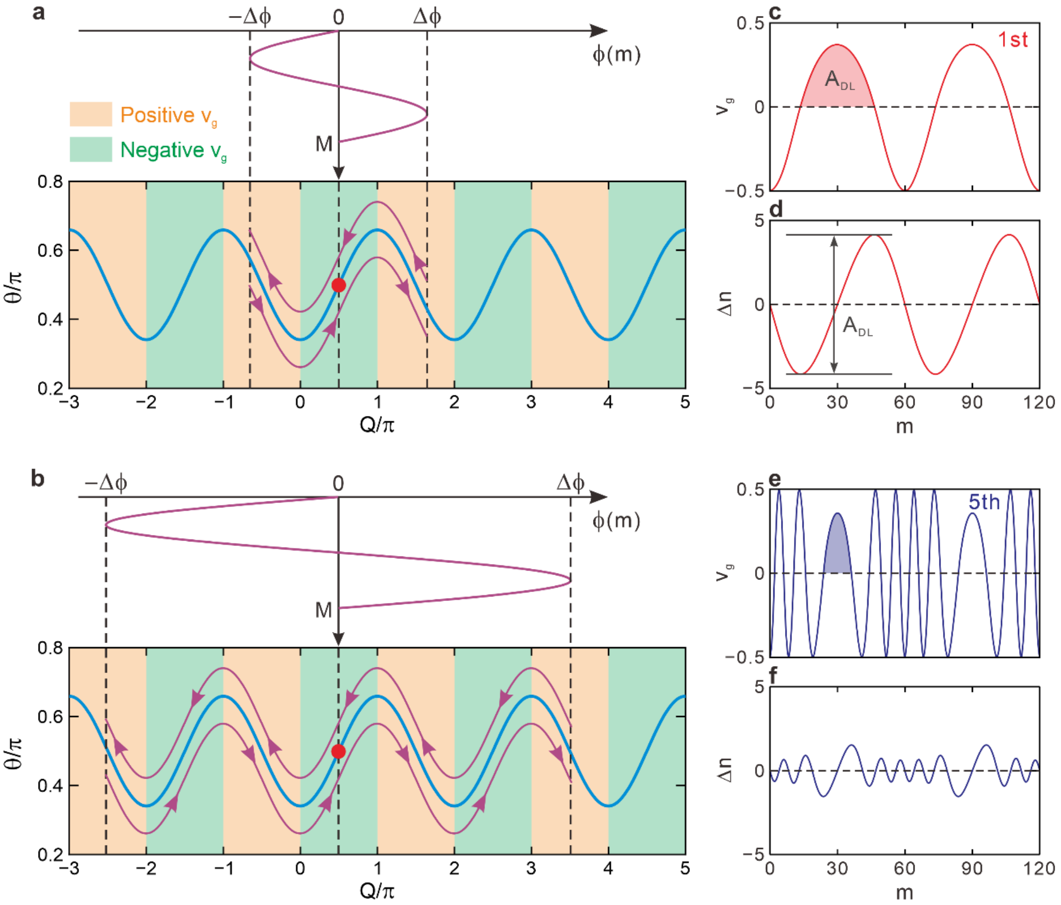

Fig. S4. Explanation for enhanced localization strength of high-order DL. a, b Schematic diagram of periodic momentum motion under weak and strong ac electric fields. The orange and green regions correspond to the positive and negative group velocities, respectively. The interference of two regions

indicates the boundary or center of Brillouin zone. **c, e** Group velocity v_g varying with step m for 1st- and 5th-order DLs. A_{DL} denotes the peak-to-peak amplitude of the wave packet oscillation, which is represented by the area of the red or blue region. **d, f** Transverse displacement Δn varying with step m for 1st- and 5th-order DLs.

More specifically, in the presence of the artificial ac electric field, the group velocity of the wave packet (belonging to the upper band) can be written as

$$v_g(m) = -\cos(\beta)\sin\left[Q - \Delta\phi\cos(\omega m + \varphi)\right]. \tag{28}$$

Figures S4c and S4e plot the group velocity $v_g(m)$ varying with step m for the 1st- and 5th-order DLs. By integrating group velocity $v_g(m)$ with respect to step m, we get the displacement of wave packet, as shown in Figs. S4d and S4f. One can see that the wave packet exhibits much more frequent oscillations during propagation for the 5th-order DL due to the faster variation of group velocity $v_g(m)$. Furthermore, the wave packet spends a shorter transport time in an individual direction, leading to a shorter transport time from the leftmost to the rightmost positions. As a consequence, the wave packet oscillates with a smaller peak-to-peak amplitude A_{DL} during the 5th-order DL, reflecting the tighter localization of wave packet.

Supplementary Note 5: Different-order DLs at long driving period

When moving from the 2nd- to higher-order DLs, the improvement of localization strength is directly proportional to the ac driving period M. In our experiment, we choose a relatively small driving period of $M = 120$, such that the localization strength enhancement is not so obvious from 2nd to 5th orders. Here we choose a relatively larger driving period $M = 400$ and numerically verify that the decrease of $\langle n^2(m)\rangle_{\max}$ can be improved. As illustrated in Fig. S5a, $\langle n^2(m)\rangle_{\max}$ decreases from 124.3 to 65.4 from the 2nd- to 4th-order DLs, indicating a significant reduction of 47%. Similar to the case of $M = 120$, when approaching the 5th-order DL, $\langle n^2(m)\rangle_{\max}$ also shows a slight increase and becomes 71.3. As also shown in Figs. S5b-d, the 4th- and 5th-order DLs exhibit more localized oscillation ranges compared to 2nd-order DL, further verifying the localization strength enhancement at a long driving period. In our current setup of fiber-loop experiment, due to the accumulated noise from optical amplifiers, it is not easy to observe 400 or even more circulation times for the optical pulse at a sufficiently low noise level. Under the circumstances, we only show the experimental results for $M = 120$ in this work.

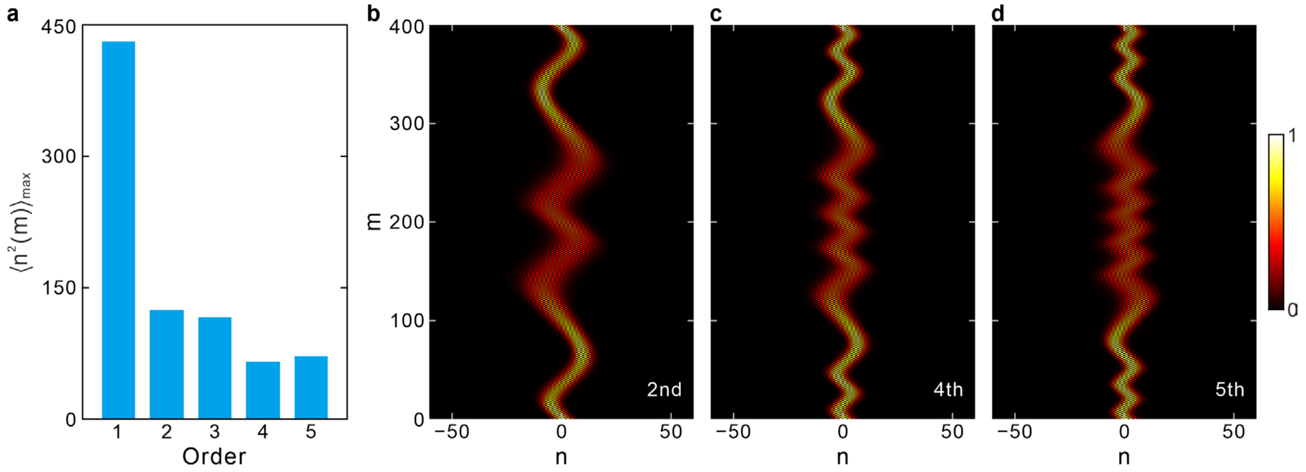

Fig. S5. a Maximum mean-square displacement $\langle n^2(m)\rangle_{\max}$ versus order of DL for driving period $M = 400$. **b-d** Simulated pulse intensity evolutions for 2nd-, 4th- and 5th-order DLs.

Supplementary Note 6: Pulse intensity evolutions for various-order DLs under external noise

We introduce external random noise into the temporal photonic lattice by imposing random phase modulation in the two coupled fiber loops, as discussed in the main text. Figure S6 illustrates the simulated and measured pulse intensity evolutions for different orders of DLs under the noise. For the 1st-order DL, the wave packet is severely disturbed by the noise, which causes the wave-packet width to be increased by 5 times. As the order increases, the wave-packet expansion decreases, reflecting that the high-order DL exhibits better robustness against the noise.

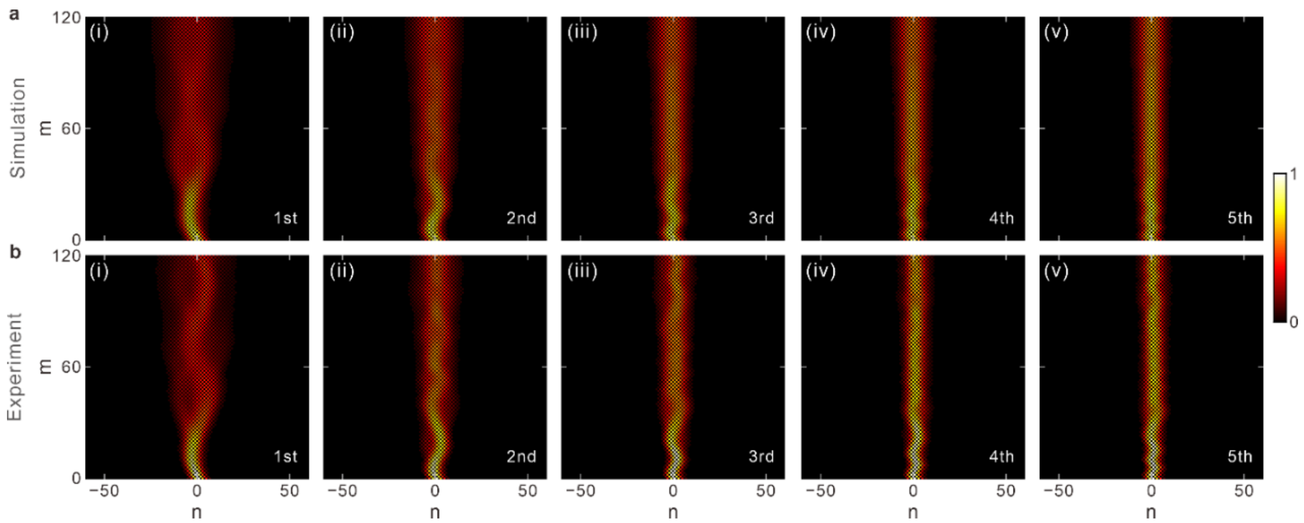

Fig. S6. Pulse intensity evolutions for various-order DLs under external noise. **a** Simulated pulse intensity evolutions for different-order DLs under external noise. (i)-(v) correspond to the DLs from the 1st to 5th orders. **b** Measured pulse intensity evolutions. (i)-(v) correspond to the simulated results in **a**(i)-**a**(v).

Supplementary Note 7: Different-order DLs under Gaussian and gamma noise

Previously, we focused on the influence of a uniformly distributed noise on high-order DLs and found that high-order DLs have much better robustness compared to low-order ones. Here we choose two other mostly-studied noise models with Gaussian and gamma distributions to further demonstrate the enhanced robustness of high-order DLs. The simulation results show that the enhanced robustness of high-order DLs is universal for different types of noise models.

Firstly, for a modulation-phase noise ϕ_{noise} with Gaussian distribution, its probability density function is given by

$$P(\phi_{\text{noise}}) = \frac{1}{\sqrt{2\pi\sigma^2}} e^{-(\phi_{\text{noise}}-\mu)^2/2\sigma^2}, \tag{29}$$

where μ and σ are the expected value and standard deviation of the random noise, respectively. As plotted in the inset of Fig. S7a, the probability density function possesses a Gaussian shape. Here we choose $\mu = 0$ and $\sigma = 0.043\pi$, both of which equal to those of the uniformly distrusted noise used in the main text. To study the influence of Gaussian noise on DLs, we simulate the wave packet dynamics under 5000 realizations of Gaussian noises and calculate the averaged inverse participation ratio (IPR) at output step $m = 120$, as depicted in Fig. S7a. One sees that the IPR versus the modulation amplitude $\Delta\phi$ is almost identical to that of a uniform noise (see Fig. 4b in the main text). The value of IPR peaks at each order of DLs, indicating where the noise-induced wave packet spreading should be suppressed. Besides, the IPR increases as the DL order increases, reflecting that the high-order DL manifests better robustness against the Gaussian noise. Figures S7b(i)-S7b(iv) illustrate the simulated pulse intensity evolutions for $\Delta\phi = 0$, 2.4 (1st-order DL), 8.7 (3rd-order DL) and 14.9 (5th-order DL), respectively. For the case without external field ($\Delta\phi = 0$), the wave packet spreads in a wide range of direction under the noise. As the DL occurs, the spreading of the wave packet is highly suppressed by comparing with the field-free case. As the order of DL increases, the suppression becomes more remarkable. Especially for the 5th-order DL, the width of wave packet almost keeps constant during evolution.

Next, we consider the gamma noise with the probability density function

$$P(\phi_{\text{noise}}) = \frac{1}{b^a\Gamma(a)} \phi_{\text{noise}}^{a-1} e^{-\phi_{\text{noise}}/b} - ab, \tag{30}$$

where a and b are two positive parameters determining the shape of gamma distribution, and $\Gamma(a)$ is the gamma function. Note that the expected value of the noise is zero, and the standard deviation equals $a^{1/2}b$. Here, we choose $a = 1.85$ and $b = 0.1$ to ensure the standard deviation of noise remains the same level with the cases of uniform and Gaussian noise. As depicted in the inset of Fig. S7c, the random phase manifests a gamma distribution. In the presence of gamma noise, we calculate the averaged

inverse participation ratio (IPR) at m = 120 in Fig. S7c. One sees clearly that the enhanced robustness of high-order DLs is also well preserved for gamma noise, and the dependence of IPR on $\Delta\phi$ is identical to these of the uniform and Gaussian cases. In Figs. S7d(i)-S7d(iv), the simulated pulse intensity evolutions also verify the above analysis. Hence, the enhanced robustness of high-order DL is universal for more realistic noise models.

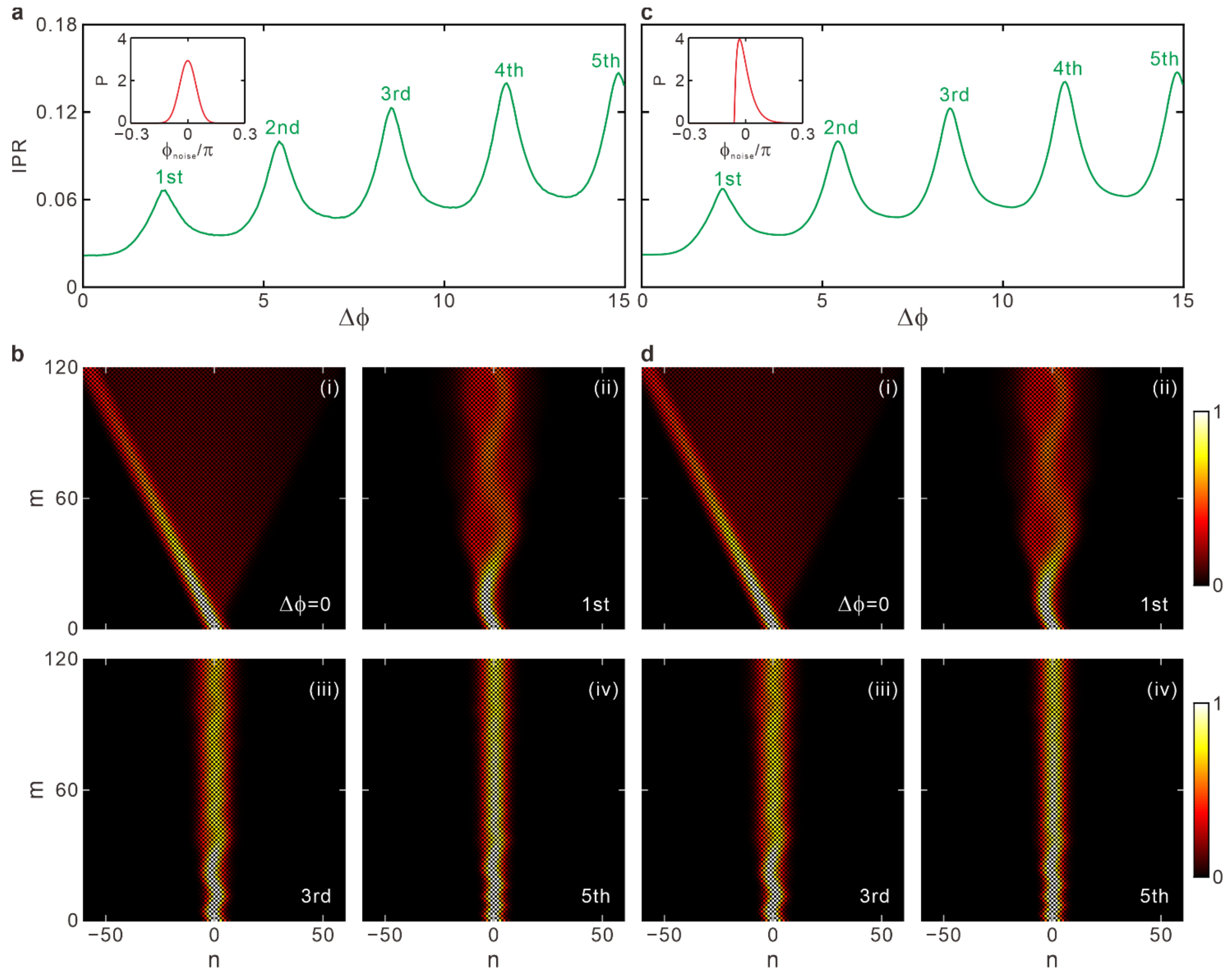

Fig. S7 a Inverse participation ratio IPR of wave packet at m = 120 in the presence of Gaussian noise. The inset displays the probability density function of Gaussian noise. **b** Simulated pulse intensity evolutions for (i) $\Delta\phi$ = 0. (ii)-(iv) The 1st, 3rd- and 5th-order DLs, respectively. **c** Inverse participation ratio IPR of wave packet at m = 120 under gamma noise. The probability density function of gamma noise is shown in the inset. **d** Simulated pulse intensity evolutions for (i) $\Delta\phi$ = 0. (ii)-(iv) The 1st, 3rd- and 5th-order DLs, respectively.

Supplementary Note 8: Tunability of cloaking window size

Here, we theoretically analyze the relationship between the cloaking window size and the driving period M. As the split wave packets enter the cloaking region with biased modulation phase ϕ_2, the transverse group velocity corresponding to the two bands reads

$$v_{g,\pm}(m) = \mp\cos(\beta)\sin\left[Q - \Delta\phi\cos(\omega m + \varphi) - \phi_2\right]. \tag{31}$$

The Bloch momentum and initial phase are chosen as $Q = \pi/2$ and $\varphi = \pi/2$, respectively. To construct a temporal cloak, the biased modulation phase outside and within the cloak region are set to $\phi_1 = 0$ and $\phi_2 = \pi/2$, respectively. As a result, the group velocity can be simplified to

$$v_{g,\pm} = \mp\cos(\beta)\sin\left[\Delta\phi\sin(\omega m)\right]. \tag{32}$$

Figure S8a plots the group velocity for lower and upper bands as $\Delta\phi = 2.4$ (corresponding to the 1st-order DL). For the lower band, the group velocity is positive at the first half of driving period M, indicating that the wave packet will move to the right continuously and reach the rightmost position at $m = M/2$. In contrast, the wave packet belonging to the upper band reaches the leftmost position at $m = M/2$. Hence, the time window has a maximum width at $m = M/2$. For the lower (upper) band, the wave-packet displacement at $m = M/2$ can be represented as the area of the blue (red) region in Fig. S8a. By adding the two areas, the maximum width, i.e., cloak width, can be written as

$$W_{\max} = \int_0^{M/2} | v_{g,-}(m) - v_{g,+}(m) | \, dm = MW_0, \tag{33}$$

in which

$$W_0 = 2\cos(\beta)\int_0^{1/2}\sin\left(\Delta\phi\sin\frac{2\pi m}{M}\right)d\left(\frac{m}{M}\right). \tag{34}$$

The parameter W_0 is a constant and equals to 0.37 as $\beta = \pi/3$. According to Eq. (33), the cloak width is in direct proportion to the driving period M. As shown in Fig. S8b, the cloak width increases linearly with the driving period M. In addition, the longitudinal length of the cloak is equal to the driving period M. Thus, we can manipulate not only the transverse width but also the longitudinal length of the cloaking window by controlling the driving period M.

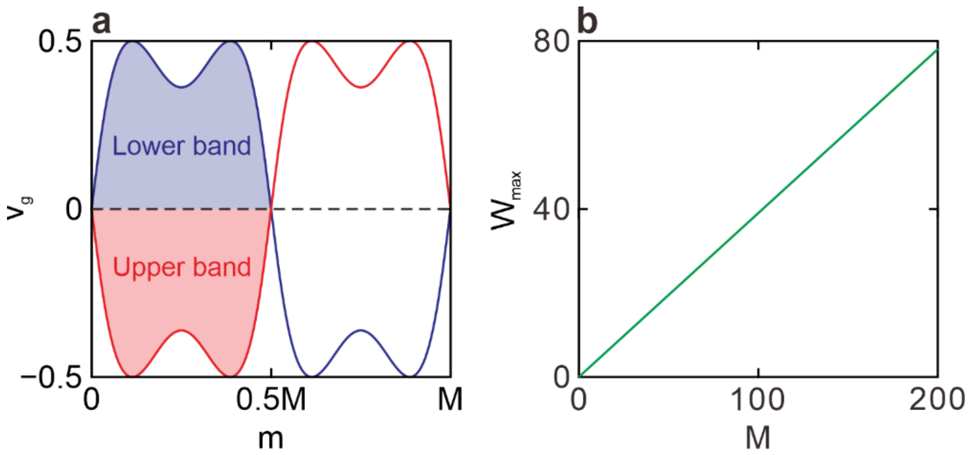

Fig. S8. Tunability of cloaking window size. a Transverse group velocity v_g versus step m for lower and upper bands. The area of blue (red) region denotes the wave-packet displacement for the lower (upper) band at $m = M/2$. **b** Cloak width $W_{\max}$ as a function of driving period M.

Supplementary Note 9: Cloak shape engineering based on ac-driving DL

In principle, the cloaking can be realized by exploiting the wave packet's self-imaging effects, such as Bloch oscillations (BOs) under a dc-field driving and our DL scheme under an ac-field driving. Based on the DL, we already showed that the cloak possesses flexible reconfigurability both in the cloaking window size and its opening time. Even more exciting, for a given fixed driving period the cloaking region can be dynamically reshaped, holding significant advantages over the cloak based on BOs. In fact, the shape of the cloaking region is basically given by the area enclosed by the paths followed by the two split beams, which can be estimated from the group velocities corresponding to two different bands

$$v_{g,\pm}(m) = \mp\cos(\beta)\sin\left[Q(m)\right] = \mp\cos(\beta)\sin\left[Q-\phi(m)\right]. \tag{35}$$

For a dc field α, leading to BOs, the momentum $Q(m) = Q+\alpha m$ changes linearly in time, so that the path followed by the beam exactly reproduces the shape of band structure. The main limits of using the BO approach is that we cannot reshape the cloaking region: its shape always reproduces the shape of the energy dispersion curve. On the other hand, using an ac field we can control and dynamically reconfigure the path of the beam, for a fixed value of the driving period. By contrast, it is known that the path followed the quantum particle in the crystal under an ac field does not reproduce the shape of the dispersion curve and can be dynamically controlled, for example, by the initial phase of the ac field. In particular, for a sinusoidal field $E_{\text{eff}}(m) = \omega\Delta\phi\sin(\omega m+\varphi)$, as in our experiment, by varying the phase φ of the field (from 0 to π) we can largely deform the beam path, i.e. reshaping the cloaking region. This is shown as an example in the Supplementary Movie 1, where we compare the beam paths under BOs (for a dc field $\alpha = \omega$) and under DL for increasing values of the phase φ. Parameter values are $\beta = \pi/3$, $\Delta\phi = 2.4$, $\omega = \pi/60$. Clearly, the movie shows that, at a fixed value of the duration time (defined by the period $M = 2\pi/\omega$), the beam path in the DL regime can be largely tailored by changing the phase of the sinusoidal field.

For a non-sinusoidal ac field, an even more flexible cloaking region reshaping could be realized. As an illustrative example, in Fig. S9 we show how the paths of wave packets (and thus the cloaking region) can be shaped by changing the ac field $\Delta\phi(m)$. The only requirement to observe self-imaging after one oscillation cycle is provided by the DL condition

$$\Delta n_\pm = \int_0^M v_{g,\pm}(m)\,dm = \mp\int_0^M \cos(\beta)\sin\left[Q-\phi(m)\right]dm = 0, \tag{36}$$

which reduces to the usual condition of $J_0(\Delta\phi)=0$ for the sinusoidal field, as demonstrated by our work. Panel (a) reproduces our experiment, where we used a sinusoidal field at first-order DL point, i.e. $\Delta\phi(m) = \Delta\phi\cos(\omega m+\varphi)$ with $\Delta\phi = 2.4$, $\varphi = \pi/2$, $\omega = \pi/60$, $\beta = \pi/3$. Panel (b) corresponds to a modified ac field shaped as $\Delta\phi(m) = \Delta\phi_1\cos(\omega m+\varphi)+\Delta\phi_2\sin^3(\omega m+\varphi)$ with $\Delta\phi_1 = 1$, $\Delta\phi_2 = 3.1$, $\varphi = 2.5$, $\omega = \pi/60$, $\beta = \pi/3$.

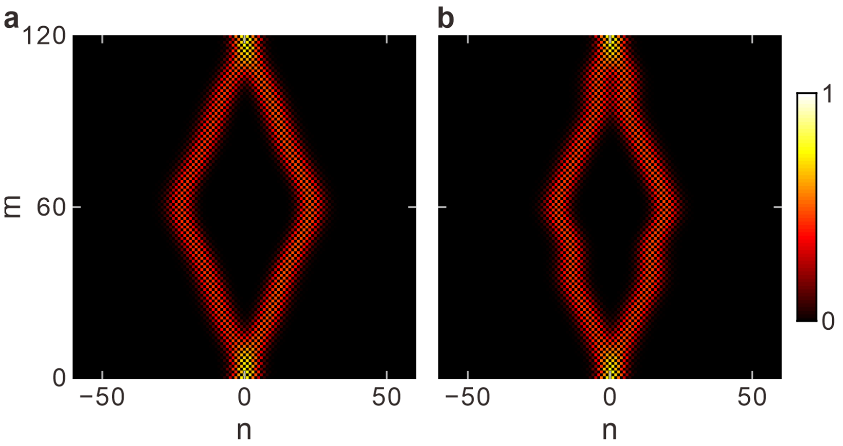

Fig. S9. Example of reconfigurability of the cloaking region in the mesh lattice setup by change of the ac field shape. In **a** $\Delta\phi(m) = \Delta\phi\cos(\omega m+\varphi)$, in **b** $\Delta\phi(m) = \Delta\phi_1\cos(\omega m+\varphi)+\Delta\phi_2\sin^3(\omega m+\varphi)$.

Supplementary Note 10: Width comparison between DL- and BO-based cloaks

Compared with the BO-based cloak, the DL-based cloak also holds the advantage in the cloak's width. For BO-based cloak under a dc field α, the transverse group velocities for lower and upper bands read

$$v_{g,\pm}(m) = \mp\cos(\beta)\sin(Q+\alpha m-\phi_2), \tag{37}$$

As $Q = \pi/2$ and $\phi_2 = \pi/2$, the group velocities reduce to

$$v_{g,\pm}(m) = \mp\cos(\beta)\sin(\alpha m). \tag{38}$$

At the first half of the Bloch period $M = 2\pi/\alpha$, the group velocities corresponding to the lower and upper bands are positive and negative, respectively. As a consequence, the wave packets belonging to the lower and upper bands continuously move to the rightward and leftward, respectively, leading to the opening of a time window. At the second half of Bloch period, the wave packets' group velocities become opposite to the first ones, and the time window is gradually closed. The maximum width of the time window, i.e. the cloak width, can be calculated as

$$W_{\max} = \int_0^{M/2}\left[v_{g,-}(m)-v_{g,+}(m)\right]dm = \frac{2\cos(\beta)}{\pi}M. \tag{39}$$

Figure S10a depicts the cloak width $W_{\max}$ with respect to the period M for BOs and DL. One sees that the widths of BO- and DL-based cloaks are both directly proportional to the oscillation period M of BOs and DL. Furthermore, for an identical oscillation period M, the DL-based cloak has a broader width, promising a better capability in practical applications. The differences in the cloak width can be explained by the group velocities of wave packets displayed in Figs. S10b and S10c. At the first half of the oscillating period, the velocity difference between two wave packets that belong to lower and upper band makes the two wave packets separated and results in the maximum separation at $m = M/2$. The cloak width could be calculated by adding the areas of blue and red regions that denote the displacements of two wave packets. For DL, the colored region is obviously larger than the one of BOs, which reflects a bigger cloak width. Figures S10d and S10e display the pulse intensity evolutions of DL- and BO-based temporal cloaks with a period of $M = 120$. The DL-based cloak has a broader width $W_{\max} = 45$ while the BO-based one corresponds a smaller cloak width of $W_{\max} = 38.2$.

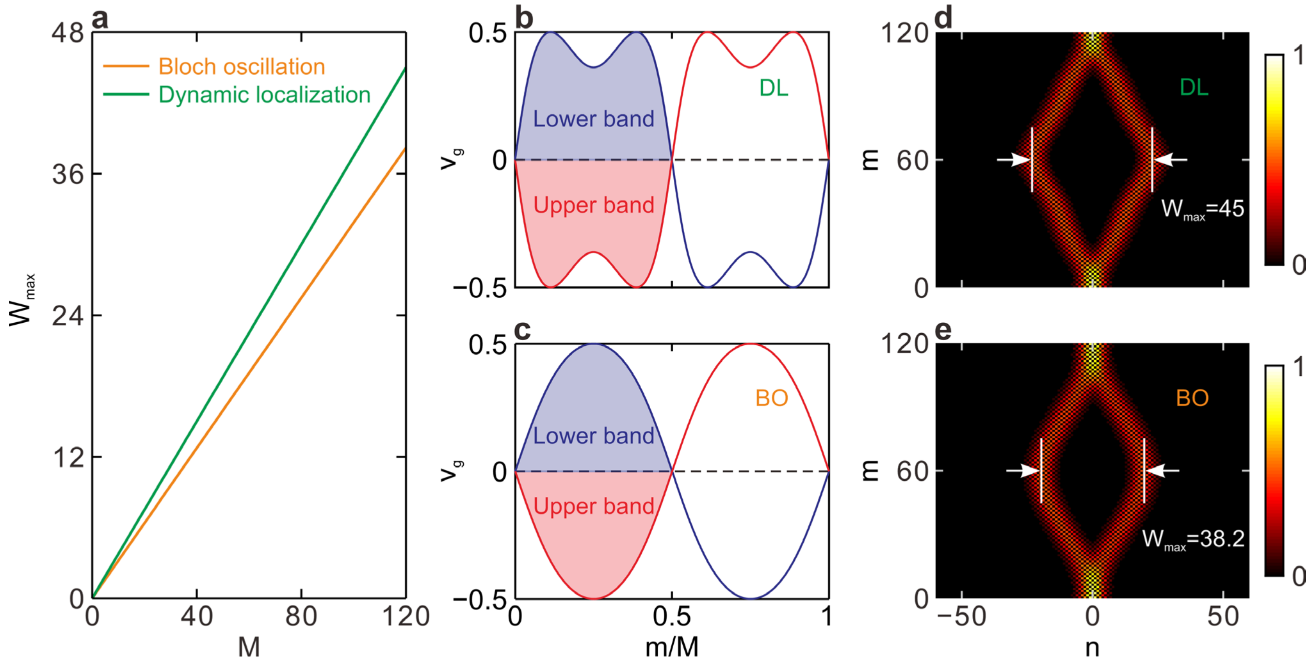

Fig. S10. **a** Cloak width $W_{\max}$ varying with period M for DL and BOs. **b**, **c** Transverse group velocity v_g versus step m for DL and BOs. The area of blue (red) region represents the displacement of wave packet for the lower (upper) band after the first half of the period M. **d**, **e** Simulated pulse intensity evolutions for DL- and BO-based temporal cloaks.
