# Supplementary material for: High-order dynamic localization and tunable temporal cloaking in ac-electric-field driven synthetic lattices
Source: Nat Commun. 2022 Dec 10;13:7653. doi: 10.1038/s41467-022-35398-9 (PMC9741653; doi:10.1038/s41467-022-35398-9)
Supplement: Supplementary file 3 — Description of Additional Supplementary Files [file 41467_2022_35398_MOESM3_ESM.pdf]

File name: Supplementary Movie 1

Description: Shows the differences between the temporal cloaks based on the dynamic localization and Bloch oscillations.
